# Supplementary material for: Lipoic Acid Combined with Melatonin Mitigates Oxidative Stress and Promotes Root Formation and Growth in Salt-Stressed Canola Seedlings (Brassica napus L.)
Source: Molecules. 2021 May 25;26(11):3147. doi: 10.3390/molecules26113147 (PMC8197368; doi:10.3390/molecules26113147)
Supplement: Supplementary file 1 [file molecules-26-03147-s001.zip › molecules-1195654-supplementary.pdf]

Supplementary Materials

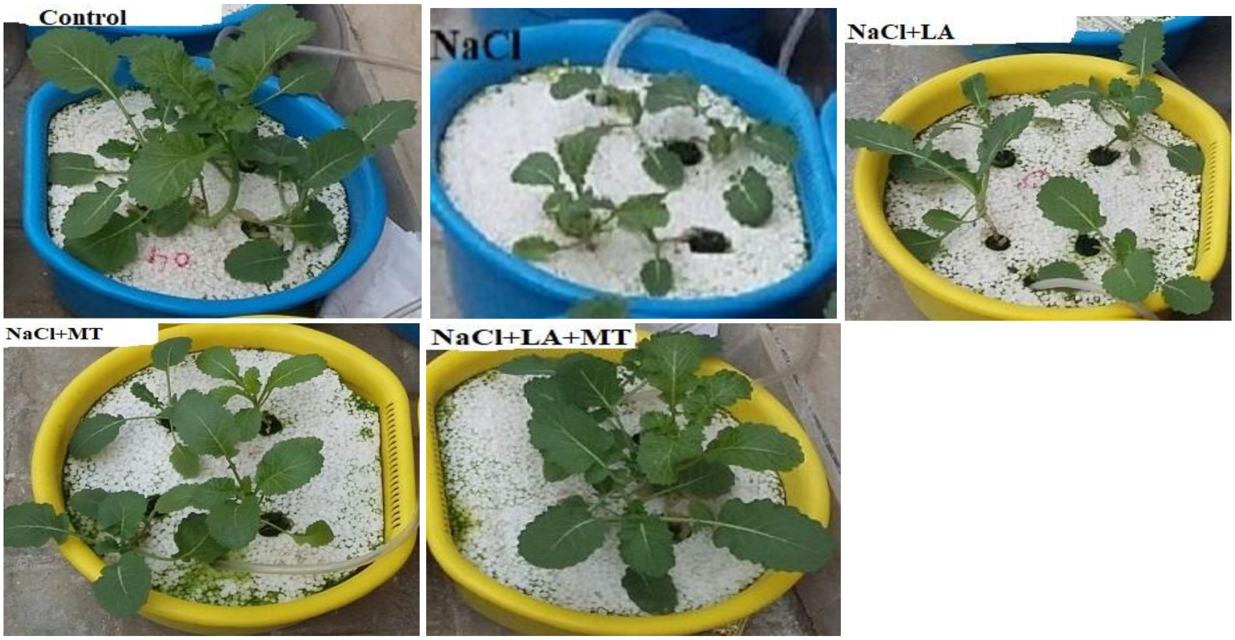

Figure S1. Influence of lipoic acid (LA) and melatonin (MT) and their combination (LA+MT) on the growth of canola seedlings under salt stress (S; 100 mM NaCl)

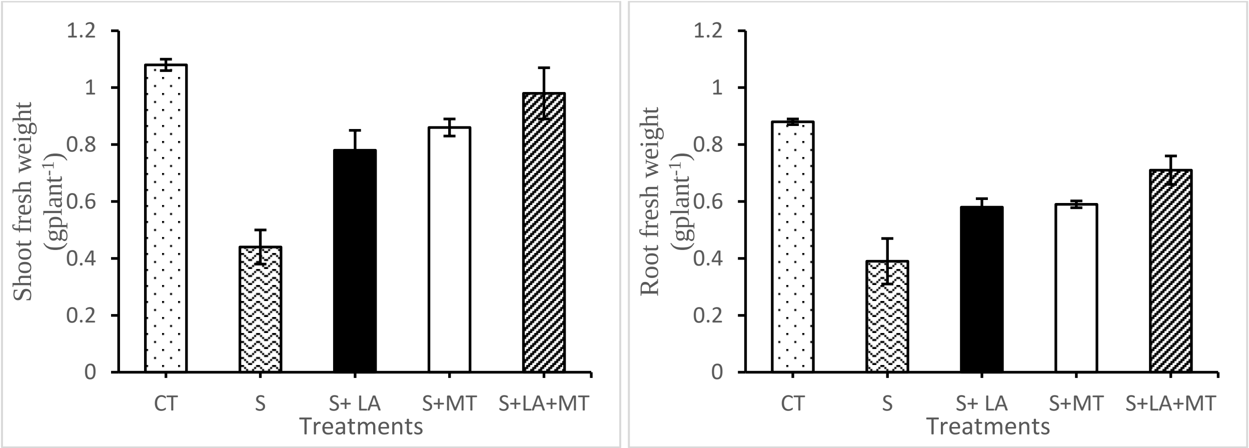

Figure S2. The influence of lipoic acid (LA), melatonin (MT) and their combination (LA+MT) on the morphology of canola seedlings under salt stress (S; 100 mM NaCl)

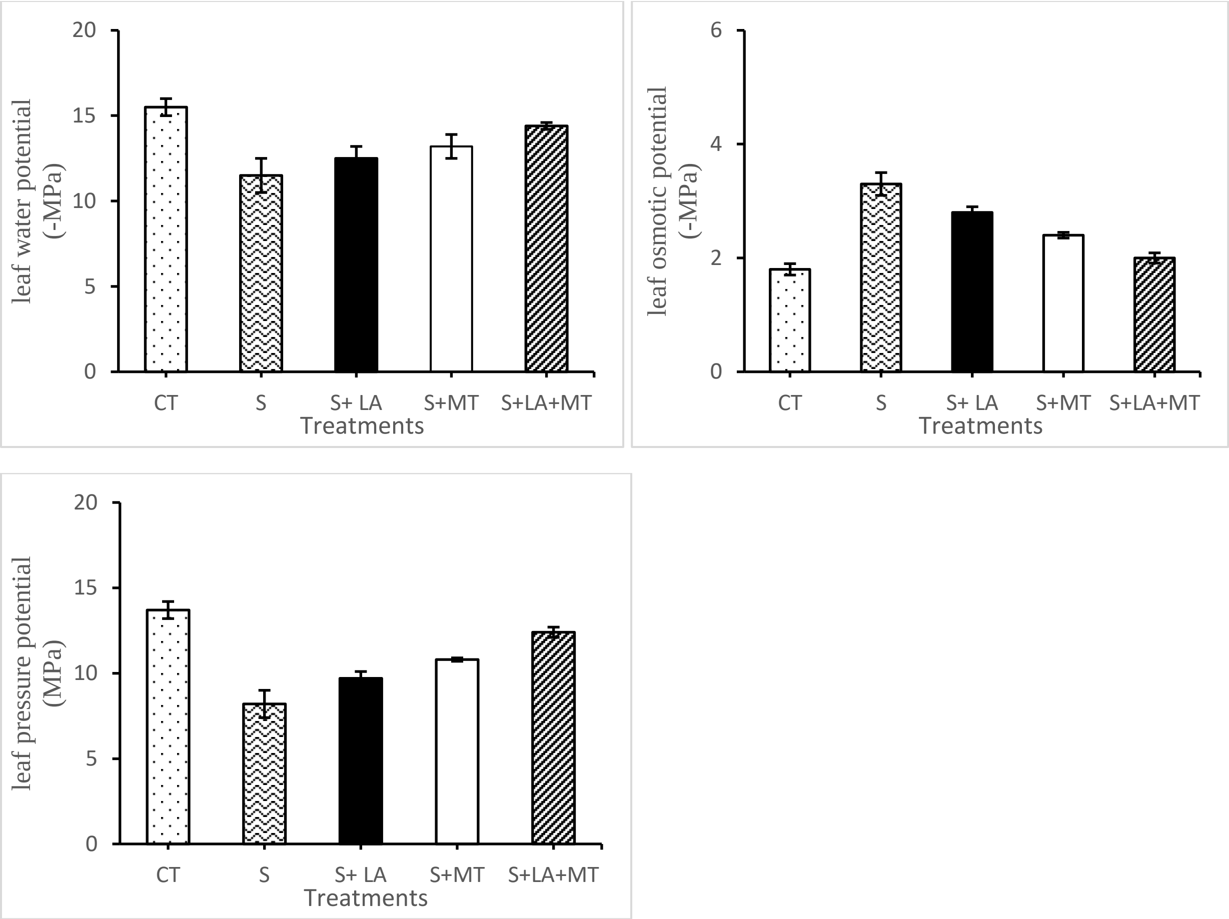

Figure S3. The influence of lipoic acid (LA), melatonin (MT) and their combination (LA+MT) on the water relation of canola seedlings under salt stress (S; 100 mM NaCl)

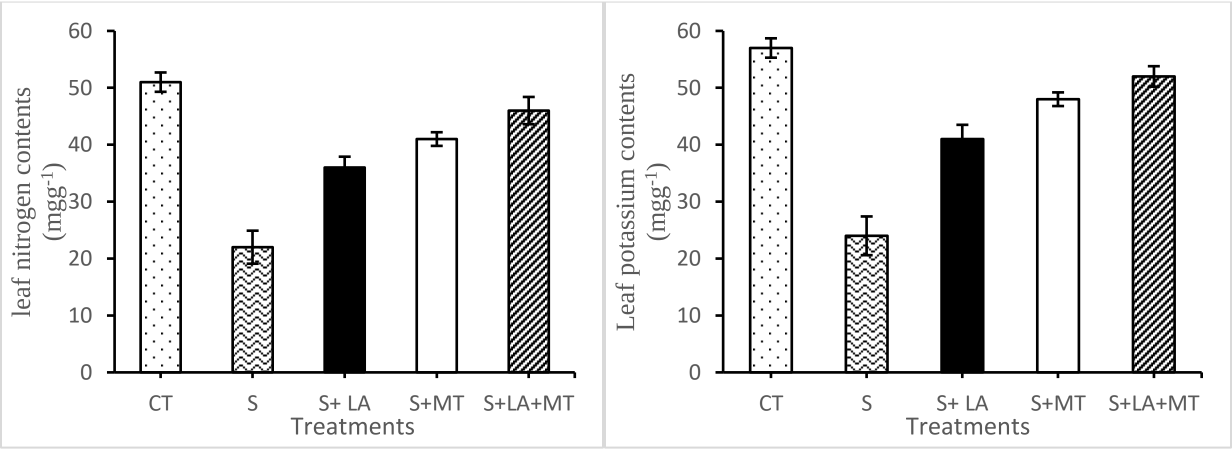

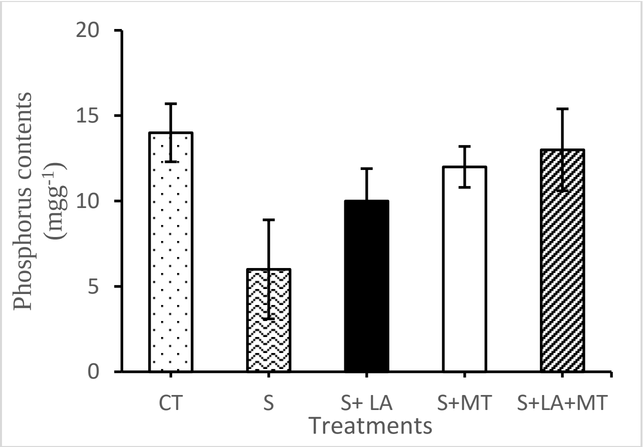

Figure S4. The influence of lipoic acid (LA), melatonin (MT) and their combination (LA+MT) on the leaf minerals composition of canola seedlings under salt stress (S; 100 mM NaCl)
